# The effect of AST/ALT (De Ritis) ratio on survival and its relation to tumor histopathological variables in patients with localized renal cell carcinoma

**DOI:** 10.1590/S1677-5538.IBJU.2017.0173

**Published:** 2018

**Authors:** Lütfi Canat, Hasan Anil Ataly, Samir Agalarov, İlter Alkan, Fatih Alturende

**Affiliations:** 1 Department of Urology, Okmeydani Training and Research Hospital, Istanbul, Turkey

**Keywords:** Prognosis, Survival, Pathology

## Abstract

**Purpose:**

To assess the relationship between De Ritis (aspartate aminotransaminase [AST]/Alanine aminotransaminase [ALT]) ratio and pathological variables and whether it is an independent prognostic factor.

**Materials and Methods:**

We analyzed 298 consecutive patients who underwent radical or partial nephrectomy for non-metastatic renal cell carcinoma (RCC) between 2006 and 2015. The association between De Ritis ratio and pathological variables including tumor size, presence of renal vein invasion, vena cava invasion, renal capsule infiltration, Gerota fascia invasion, renal sinus involvement, renal pelvic invasion, angiolymphatic invasion, adrenal gland involvement, lymph node involvement, tumor necrosis, and Fuhrman's grade was tested. Multivariable Cox analysis was performed to evaluate the impact of this ratio on overall survival and cancer-specific survival.

**Results:**

An increased preoperative De Ritis ratio was significantly associated with renal vein invasion, renal capsule infiltration and renal pelvis involvement (p<0.05) in non-metastatic RCC. On multivariate analysis we found that tumor size, Fuhrman grade and lymph node involvement were independent prognostic factors for cancer-specific survival. AST/ALT ratio had no influence on the risk of overall and cancer-specific survival.

**Conclusion:**

An increased preoperative AST/ALT ratio had a significant association with renal vein invasion, renal capsule infiltration and renal pelvis involvement in patients with non-metastatic RCC. However, it does not appear to be an independent prognostic marker in non-metastatic RCC.

## INTRODUCTION

Renal cell carcinoma (RCC) accounts for 3% of all malignancies and thanks to advanced radiological tools, the incidence of RCC has increased during the last decade (1, 2). It is important to research useful prognostic markers for the prognosis of RCC patients. Several prognostic parameters have been evaluated; stage, histologic type Fuhrman grade, several risk scores and nomograms remain crucial prognostic factors in RCC (3-5). Even though some of these prognostic models showed predictive accuracy, the correctness of prognostic factors should be demonstrated by novel research.

Aminotransaminases are expressed in several cellular compartments by malignant or nonmalignant cells. Alanine aminotransaminase (ALT) is only existent in the hepatocellular cytoplasm and mitochondria; however, aspartate aminotransaminase (AST) is widely spreaded in several organs, including heart, kidney, brain, skeletal muscle, and liver (6). The relationship between different levels of these enzymes and patient prognosis is stated in several types of cancer, previously (7-9).

Fernando De Ritis described the ratio of AST and ALT firstly, in 1957 and this ratio has been known ever since as the De Ritis ratio (10). Previous studies have demonstrated that this ratio to be crucial prognostic factors in different malignancies (11-13). Bezan et al. and Lee et al. reported that this ratio can predict survival outcomes in patients with non-metastatic RCC and they found that an elevated AST/ALT ratio was significantly associated with inferior survival outcomes (14, 15). The aim of our study was to evaluate the effect of AST/ALT ratio on prognosis in our center and to investigate the relationship between this ratio and all histopathological variables in patients with surgically treated for non-metastatic RCC.

## MATERIALS AND METHODS

In this retrospective analysis, the medical records of 412 patients who underwent radical or partial nephrectomy for non-metastatic RCC at our department between 2006 and 2015 were reviewed. The study was approved by the local committee of ethics and informed consents were obtained from eligible patients.

The databases included the basic demographic features, preoperative laboratory parameters, pathological findings and survival of patients. Plasma AST and ALT levels were analyzed by standard clinical methodology within 7 days prior to surgery. Pathological characteristics included tumor size, presence of renal vein invasion, vena cava invasion, renal capsule infiltration, Gerota fascia invasion, renal sinus involvement, renal pelvic invasion, tumor necrosis, angiolymphatic invasion, adrenal gland involvement, and lymph node involvement. Cellular grading was performed by Fuhrman's grading system (16) and staging was classified according to TNM 2010 classification (17). The pathological assessment was performed by an experienced pathologist.

Patients with distant metastasis (n=42) prior to treatment, patients with chronic hepatitis (n=9), other malignancies (n=8), pre-treatment or post-treatment chemotherapy, radiotherapy or immunotherapy (n=22), mixed type RCC or familial RCC (n=8), and incomplete information (n=25) were excluded from the study. The remaining 298 patients were included.

Postoperatively, every 6 months, all patients were assessed for the first 2 years and yearly thereafter. Cancer-specific survival (CSS) and overall survival (OS) rates were defined at the last follow-up date using surgery date to the date of cancer-specific mortality and any-cause of mortality. The pathological findings and their relation with the AST/ALT ratio were evaluated to determine which pathological parameters is affected by AST/ALT ratio. Since the optimum cut-off level of AST/ALT ratio of 1.5 had maximum Youden index value (12, 15), we also analyzed the relationship between pathological parameters and high (AST/ALT≥1.5) or low (AST/ALT<1.5) levels of this ratio.

The data was analyzed with Statistical Package for Social Sciences (SPSS) version 22 (IBM Co., Armonk, NY, USA). Comparison between the AST/ALT ratio and several histopathological parameters was assessed by the Mann-Whitney U test and chi-square test. Univariate and multivariate logistic regression tests were used to analyze the associations between preoperative variables and adverse pathologic events. CSS and OS were estimated by Kaplan-Meier method with differences evaluated by log-rank test. Multivariate Cox proportional hazard analysis were performed to detect the effect of potential confounders, including age, tumor stage, and pathological variables. Correlations were considered statistically significant at a p<0.05 level.

## RESULTS

The clinicopathological characteristics of all the patients are listed in Table-1. The patient's median age was 61 years (61.5±13.2) at surgery. 183 patients were male and 115 of them were female. The mean follow-up period was 37.8±22.3 months (17-54). Partial and radical nephrectomy was performed in 118 (39.6%) and 180 (60.4%), respectively. The histological cell types of RCC were clear cell in 210 patients (70.5%), papillary in 38 patients (12.7%), chromophobe in 24 patients (8.1%), collecting duct cell carcinoma in 5 patients (1.6%), and undifferentiated in 21 patients (7.1%). Distribution of tumor stage (I-II and III-IV) in this study was 222 (74.5%) and 76 (25.5%), respectively. Nuclear grading according to the Fuhrman classification was G1 in 18 (6.1%), G2 in 128 (42.9%), G3 in 78 (26.2%), G4 in 30 (10.1%), and undefined in 44 (14.7%). Median tumor size was 5cm (1.2-17.0cm) and median AST/ALT ratio was 1.1 (0.5-5.3).

**Table 1:** Clinicopathological characteristics of patients.

Characteristics		Min-Max	Median	Mean±s.d./n-%
Age		22-86	61	61.5±13.2
**Sex**				
	Male			183-61.4%
	Female			115-38.6%
**Histological type**				
	Clear cell			210-70.5%
	Papillary			38-12.7%
Chromophobe				24-8.1%
Collecting duct cell carcinoma				5-1.6%
Undifferentiated				21-7.1%
**T Stage**				
I-II				222-74.50%
III-IV				76-25.50%
**Fuhrman Grade**				
	I			18-6.1%
	II			128-42.9%
	III			78-26.2%
	IV			30-10.1%
	Undefined			44-14.7%
Tumor size (cm)		1.2-17.0	5.0	5.6±2.9
AST		10.0-85.0	19.0	21.8±10.7
ALT		5.0-96.0	17.0	21.5±14.8
AST/ALT		0.5-5.3	1.1	1.2±0.6

**AST =** aspartate transaminase; **ALT =** alanine transaminase

Even though comparison of prognostic factors revealed several significant results on univariate analysis, AST/ALT ratio was not a prognostic factor for cancer-specific survival (p=0.293) and overall survival (p=0.456). On multivariate analysis we found that tumor size, Fuhrman grade and lymph node involvement were independent prognostic factors for CSS (Table-2).

**Table 2:** Univariate and multivariate Cox regression models of clinicopathological characteristics for the prediction of cancer specific survival.

	Univariate Model			Multivariate Model		
	HR	%95 CI	p	HR	%95CI	p
Age	1.008	0.98 - 1.04	0.640			
Sex	5.992	1.40 - 25.73	0.016			
Histology type	0.490	0.21 - 1.16	0.106			
Fuhrman Grade	3.546	2.03 - 6.20	0.000	2.730	1.56 - 4.79	0.000
Calcium	1.571	0.71 - 3.49	0.268			
Hemoglobin	0.761	0.61 - 0.95	0.015			
Tumor size	1.308	1.17 - 1.46	0.000	1.230	1.07 - 1.41	0.003
Renal vein invasion	4.966	1.44 - 17.09	0.011			
Renal capsule infiltration	3.557	1.38 - 9.18	0.009			
Renal sinüs involvement	4.836	2.04 - 11.49	0.000			
Adrenal gland involvement	6.332	1.47 - 27.32	0.013			
Gerota fascia invasion	35.738	3.99 - 319.8	0.001			
Lymph node involvement	13.970	4.62 - 42.22	0.000	6.716	2.16 - 20.9	0.001
Angiolymphatic invasion	10.066	3.99 - 25.42	0.000			
Renal pelvis involvement	2.957	1.08 - 8.09	0.035			
AST	0.979	0.93 - 1.03	0.409			
ALT	0.958	0.91 - 1.00	0.073			
AST/ALT	1.306	0.79 - 2.15	0.293			

**AST =** aspartate transaminase; **ALT =** alanine transaminase; **HR =** hazard ratio

Analyzing AST/ALT ratio for any significant association with the histopathological variables, patients with renal vein invasion (n=18), renal capsule infiltration (n=34), and renal pelvis involvement (n=30) were found to have a significantly higher AST/ALT ratio (p=0.013, p=0.049, p=0.004, respectively). No other histopathological variable showed a significant association with AST/ALT ratio (Table-3). Regarding histopathological variables, patients in the high AST/ALT (AST/ALT≥1.5) group were more likely to have renal vein invasion (p=0.025), renal capsule infiltration (p=0.015), and renal pelvis involvement (p=0.001) compared with patients in the low AST/ALT (AST/ALT<1.5) group. Results of analysis showed that the level of AST/ALT was not associated with the tumor histological subtypes and pathological stage (Table-4).

**Table 3:** Association between AST/ALT ratio and histopathological variables.

		AST/ALT			P*
		Min-Mak	Medyan	Ort.±s.s.	
Renal vein invasion	(-)	0.45 - 5.31	1.06	1.20 ± 0.58	0.013
	(+)	1.10 - 2.33	1.44	1.58 ± 0.41	
Renal capsule infiltration	(-)	0.47 - 5.31	1.06	1.20 ± 0.58	0.049
	(+)	0.45 - 2.60	1.40	1.41 ± 0.54	
Renal sinüs involvement	(-)	0.45 - 5.31	1.00	1.19 ± 0.59	0.079
	(+)	0.47 - 2.60	1.22	1.34 ± 0.53	
Adrenal gland involvement	(-)	0.45 - 5.31	1.08	1.22 ± 0.58	0.749
	(+)	1.00 - 1.40	1.10	1.17 ± 0.21	
Gerota fascia invasion	(-)	0.45 - 5.31	1.08	1.22 ± 0.58	0.505
	(+)	1.40 - 1.40	1.40	1.40 ±.	
Lymph node involvement	(-)	0.45 - 5.31	1.07	1.22 ± 0.58	0.467
	(+)	0.79 - 2.00	1.33	1.36 ± 0.53	
Angiolymphatic invasion	(-)	0.45 - 5.31	1.07	1.21 ± 0.59	0.355
	(+)	0.76 - 2.38	1.16	1.31 ± 0.52	
Renal pelvis involvement	(-)	0.45 - 2.71	1.06	1.16 ± 0.46	0.004
	(+)	0.81 - 5.31	1.56	1.74 ± 1.08	
Tumor necrosis	(-)	0.47 - 5.31	1.07	1.22 ± 0.60	0.726
	(+)	0.45 - 2.29	1.20	1.24 ± 0.50	

* Mann-Whitney u test, **AST =** aspartate transaminase; **ALT =** alanine transaminase

**Table 4:** Association of AST/ALT ratio with pathological parameters.

		AST/ALT≥1.5 n=80		AST/ALT<1.5 n=218		P
		n	%	n	%	
Renal vein invasion		8	10	10	4.58	0.025
Renal capsule infiltration		16	20	18	8.25	0.015
Renal sinüs involvement		18	22.5	42	19.26	0.325
Adrenal gland involvement		2	2.5	6	2.75	0.438
Gerota fascia invasion		2	2.5	4	1.83	0.168
Lymph node involvement		4	5	8	3.66	0.315
Angiolymphatic invasion		10	12.5	26	11.92	0.521
Renal pelvis involvement		18	22.5	12	5.5	0.001
Tumor necrosis		14	17.5	32	14.67	0.425
**Pathological stage**						**0.735**
	I-II	56	70	166	76.2	
	III-IV	24	30	52	23.8	
**Fuhrman grade**						**0.065**
	I	4	5	14	6.4	
	II	34	42.5	94	43.11	
	III	24	30	54	24.7	
	IV	12	10	18	8.25	
**Histological type**						**0.455**
	Clear-cell	58	72.5	152	69.7	
	Papillary	9	11.2	29	13.3	
Chromophobe		6	7.5	18	8.2	
Collecting duct cell carcinoma		2	2.5	3	1.3	
Undifferentiated		5	6.2	16	7.3	

**AST =** aspartate transaminase; **ALT =** alanine transaminase

## DISCUSSION

Although the De Ritis ratio was not associated with overall and cancer-specific survival for non-metastatic RCC in the present study, we found that patients with the high AST/ALT ratio were more likely to have renal vein invasion, renal capsule infiltration, and renal pelvis involvement compared with patients in the low AST/ALT ratio. We also found that tumor size, Fuhrman grade and lymph node involvement were independent prognostic factors in patients with non-metastatic RCC.

Plasma AST level and AST/ALT ratio were defined previously as a convenient markers for hepatocellular carcinoma and they were associated with poor prognosis (18, 19). Additionally, some previous studies have demonstrated that this ratio to be a significant prognostic factor in several malignancies (11, 12, 20, 21). Recently, two similar studies reported that an elevated AST/ALT ratio was significantly associated with poor survival outcomes which were not in line with our results (14, 15). Moreover, the previous studies were lacking the evaluation of the association of AST/ALT ratio with detailed histopathological variables. Up to our study, there were no other study focusing on the relationships between De Ritis ratio and a comprehensive histopathological analysis in the evaluable literature.

While ALT is considered more liver specific, AST is present in many tissue types. The functions of both these enzymes represent crucial metabolic interactions between protein and carbohydrate metabolism. They are also important in all cells that have a high metabolic activity and AST is even more vital for aerobic glycolysis (6). Hsu and Sabatini reported that malignant cells show a higher rate of aerobic glycolysis than nonmalignant cells (22). Von Hippel-Lindau (VHL) gene is known to be the most frequent mutation in RCC which is connected to increased glycolysis (23). It may be explained that AST is involved in the glycolysis pathway in RCC with the lack of a functional VHL gene. The VHL gene mutation in clear cell RCC plays an important role in activate HIF/VEGF pathway. However, our results showed that the tumor histological subtypes were not associated with AST/ALT ratio.

In our study, elevated AST/ALT ratio in the preoperative period was associated with renal vein invasion. Venous invasion is a poor prognostic factor and these tumors are associated with poor survival rates (24, 25). Regarding CT staging of segmental or main renal vein invasion, sensitivity of 59-69% have been reported (26). Therefore, there is a requirement for predicting the probability of renal vein invasion with new markers. AST/ALT ratio may be considered a valid biomarker for the prediction of renal vein invasion in decision making of the management of disease.

RCCs generally do not have a true histologic capsule, but are surrounded by a pseudo-capsule. Pseudo-capsule formation is the result of tumor growth and necrosis of the adjacent renal parenchyma (27). Jeong et al. reported that capsular infiltration appears to have a poor prognosis than those with equivalently staged RCC without capsular infiltration (28). A pseudo-capsule was detected in 26% of neoplasms on CT, in 67% of neoplasms on angiography, and in 93% of tumors on T2-weighted sequences on magnetic resonance imaging (29). For this reason, preoperative AST/ALT ratio may be a good marker for predicting renal capsular infiltration in non-metastatic RCC.

Terrone et al. reported that urinary collecting system involvement does not affect the outcome of RCC in an independent manner; however, in organ-confined tumors urinary collecting system involvement should be taken into account when planning treatments (30). We also found that renal pelvis involvement was not an independent prognostic factor for cancer-specific survival in RCC. Moreover, we demonstrated that renal pelvis involvement was associated with elevated AST/ALT ratio. This ratio should be taken into account when planning especially partial nephrectomy and follow-up.

Limitations of our study are the retrospective design with relatively few patients and the limited follow-up. Before the De Ritis ratio can be applied generally, it must be validated in large, prospective studies.

## CONCLUSIONS

An increased De Ritis ratio had a significant association with renal vein invasion, renal capsule infiltration and renal pelvis involvement in patients with non-metastatic RCC. Despite the relationship, this ratio did not represent an independent prognostic factor with respect to overall and cancer-specific survival in patients with non-metastatic RCC.

## Abbreviations

RCC: renal cell carcinoma
ALT: alanine aminotransaminase
AST: aspartate aminotransaminase
OS: overall survival
CSS: cancer specific survival
VHL: Von Hippel-Lindau
